# Induced cell toxicity originates dendritic cell death following magnetic hyperthermia treatment

**DOI:** 10.1038/cddis.2013.121

**Published:** 2013-04-18

**Authors:** L Asín, G F Goya, A Tres, M R Ibarra

**Affiliations:** 1 Instituto de Nanociencia de Aragón (INA), University of Zaragoza, Mariano Esquillor s/n, 50018 Zaragoza, Spain; 2 Departamento de Física de la Materia Condensada, Facultad de Ciencias, University of Zaragoza, 50009 Zaragoza, Spain; 3 Departamento de Oncología, Hospital Universitario Lozano Blesa, 50009 Zaragoza, Spain

**Keywords:** magnetic hyperthermia, dendritic cell, magnetic nanoparticles, toxicity

## Abstract

Magnetic hyperthermia (MH) is based on the use of magnetic nanoparticles (MNPs) to selectively increase the temperature of MNP-loaded target tissues when applying an alternating magnetic field (AMF) in the range of radiofrequency. To date, all MH research has focused on heat generation in an attempt to elucidate the mechanisms for the death of MNP-loaded cells submitted to AMF. However, recent *in vitro* studies have demonstrated the feasibility of inducing dramatic cell death without increasing the macroscopic temperature during AMF exposure. Here, we show that the cell death observed following AMF exposure, specifically that of MNP-loaded dendritic cells (DCs) in culture, was caused by the release of toxic agents into the cell culture supernatants and not due to a macroscopic temperature increase. We performed MH *in vitro* experiments to demonstrate that the supernatant of the cell culture following AMF exposure was highly toxic when added to control unloaded DCs, as this treatment led to nearly 100% cell death. Therefore, our results demonstrate that heat is not the only agent responsible for triggering cell death following MH treatment. This finding offers new perspectives for the use of DCs as the proverbial Trojan horse to vectorise MNPs to the target tumour area and these results further support the use of DCs as therapeutic agents against cancer when submitted to AMF. Furthermore, this discovery may help in understanding the mechanism of cell death mediated by exposure to AMF.

Magnetic hyperthermia (MH) selectively heats target tissues previously loaded with magnetic nanoparticles (MNPs) by applying an alternating magnetic field (AMF). In broad terms, the procedure involves the dispersion of MNPs throughout the target tissue, and the magnetic field must be properly tuned in amplitude and frequency to heat the MNPs. The physical mechanisms by which MNPs absorb energy from the AMF and dissipate it to the media in terms of heat transfer are currently under investigation. Two primary mechanisms have been proposed based on either Brownian rotation or Néel relaxation.^1^ Both Brownian and Néel relaxation processes may be present in a ferrofluid, but recent theoretical considerations and some experimental results suggest that the phenomenology observed can be explained in the framework of the Stoner–Wohlfarth model for single-domain MNPs.^2^ It has been repeatedly demonstrated that MNPs are able to couple to an AMF and subsequently absorb energy and generate heat. However, discussion remains on the potential nanoheating effect on MNP-loaded cells/tissues, which in turn could provoke the temperature increase required to trigger cell death.^3^ Therefore, the MH field remains unclear concerning the intercellular mechanisms mediating MNP-loaded cell death following AMF exposure and if heat generation is significant enough to mediate substantial levels of cell death. Herein, we present our findings concerning novel potential mechanisms responsible for cell death resulting from AMF exposure.

*In vitro* experimental results in the literature reflect the different approaches that have been used to evaluate temperature increase during AMF exposure. Some studies have demonstrated cancer cell death due to increased temperatures of 43–45 °C following AMF exposure,^4, 5^ whereas other studies have demonstrated cell death without observing any temperature increase during AMF exposure.^6, 7, 8^ In previous studies, our group demonstrated that tuning various experimental parameters could modulate the level of cell damage.^7^ Our experimental setup for these *in vitro* studies enabled precise measurements of frequency, magnetic field amplitude and MNP concentration to control for heat generation. As we have identified a set of experimental parameters that are able to produce cell death even in the absence of temperature increases in the cell culture medium, we believe that additional mechanisms besides heat must be involved in the triggering of cell death in MH.

## Results and Discussion

Dendritic cells (DCs) take part in a wide repertoire of immune responses,^9^ and because these cells possess a large capacity for incorporating antigens, microbes, microparticles and nanoparticles (NPs), they can be used as the proverbial Trojan horse to vectorise MNPs to a target tumour area.^10, 11^ We previously established that DCs in the absence of MNPs were not affected by AMF. However, under AMF, MNP-loaded DCs submitted to an AMF of 260 kHz and 12.7 kA/m and *t*_app_ of 30 min showed almost 100% cell death in the absence of any increase in temperature.^12^ In this study, we sought to understand the mechanism of cell death produced by the application of an electromagnetic field without increases in the temperature of the medium. The uptake of NPs by the DCs was induced by incubation in cell culture medium with increasing concentrations of MNPs.

**Figure 1:**
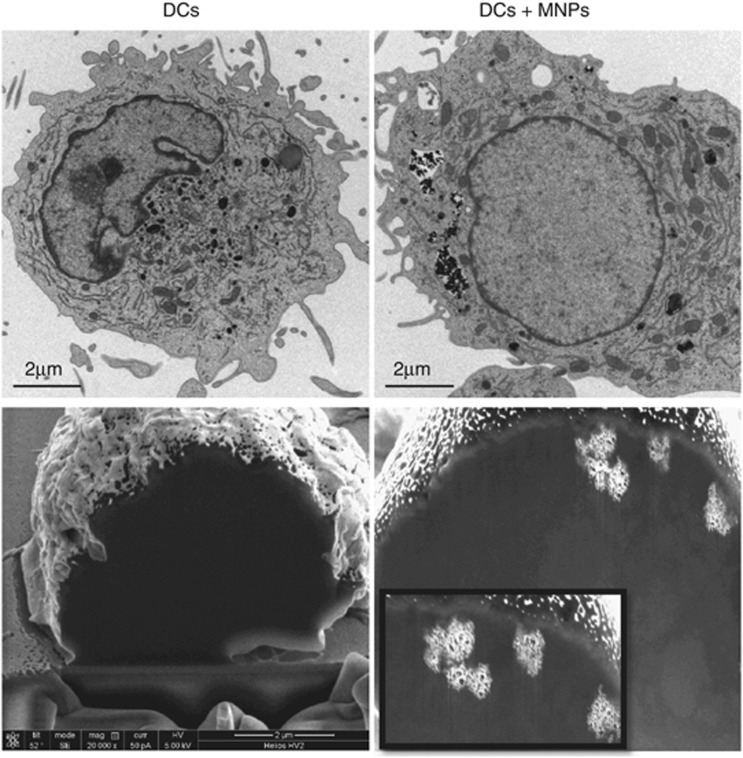
TEM (upper images) and dual beam etched micrographs (lower images) of DCs without MNPs as the reference sample and DCs incubated with MNPs at 50 *μ*g Fe_3_O_4_/ml. MNPs can be observed, as black particles in TEM images and as bright particles in dual beam images, within endocytic vesicles in the cytoplasmatic region

As shown in Figure 1, Transmission Electron Microscopy (TEM) and dual beam micrographs demonstrated that MNPs were located in the cytoplasmic region of DCs inside vesicles 1–2 *μ*m in size, which indicated that the mechanism of MNP uptake was likely phagocytosis or macropinocytosis. In both of these internalisation mechanisms, due to vesicular trafficking, the MNPs may be confined to lysosomes.

**Figure 2:**
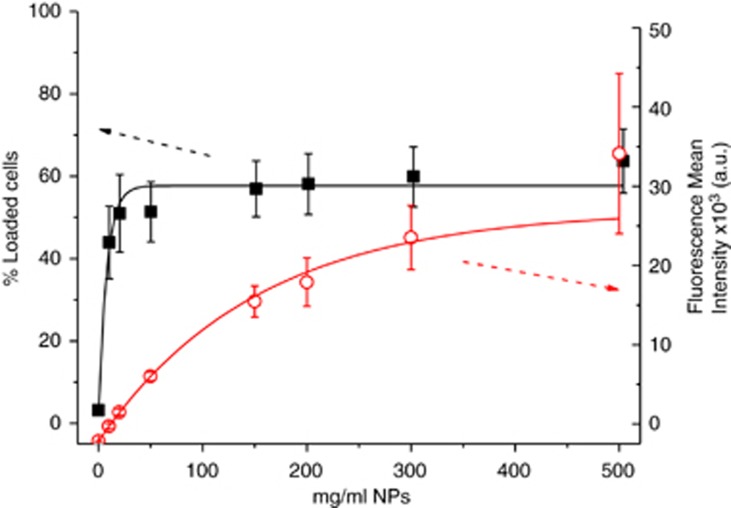
(Red open circles) Plot of the percentage of cells that internalised rhodamine (Rho)-NPs as a function of the NP incubation concentration. (Black solid squares). Representation of the fluorescence mean intensity of the population of DCs that internalised Rho-NPs *versus* NPs at each incubation concentration related to the number of NPs internalised per cell. Measurements were made by FACS. *N*=3

Notably, not all cell populations were shown to have taken up MNPs. To quantify the amount of internalised NPs and percentage of cells that had taken up NPs, fluorescent NPs were used for flow cytometric analysis. As shown in Figure 2, for concentrations of MNPs >20 *μ*g/ml, the percentage of DCs that positively incorporated the NPs did not change significantly, and this occurred even when the concentration of NPs was increased by more than 20-fold. However, when we analysed the fluorescence mean intensity, which was related to the amount of NPs internalised per cell, we observed that incubations with higher concentrations of NPs led to a greater amount of internalised NPs. These results indicate that only 50–60% of the cells were able to internalise the NPs and that the final uptake was dose-dependent. However, as a large fraction of the target cell population failed to incorporate MNPs, it was surprising that we observed almost 100% cell death following AMF exposure. Thus, we investigated the underlying mechanism responsible for the death of cells that did not contain MNPs.

We hypothesised that MNP-loaded DCs submitted to an AMF may release toxic agents into the supernatant that could trigger the death of neighbouring cells. We proposed that when MNP-loaded DCs were exposed to an AMF, a massive and sudden disruption of the lysosomes containing the MNPs may have triggered the death of those cells and subsequent release of lysosomal content into the extracellular space, which in turn could have triggered the death of the surrounding cells.

**Figure 3:**
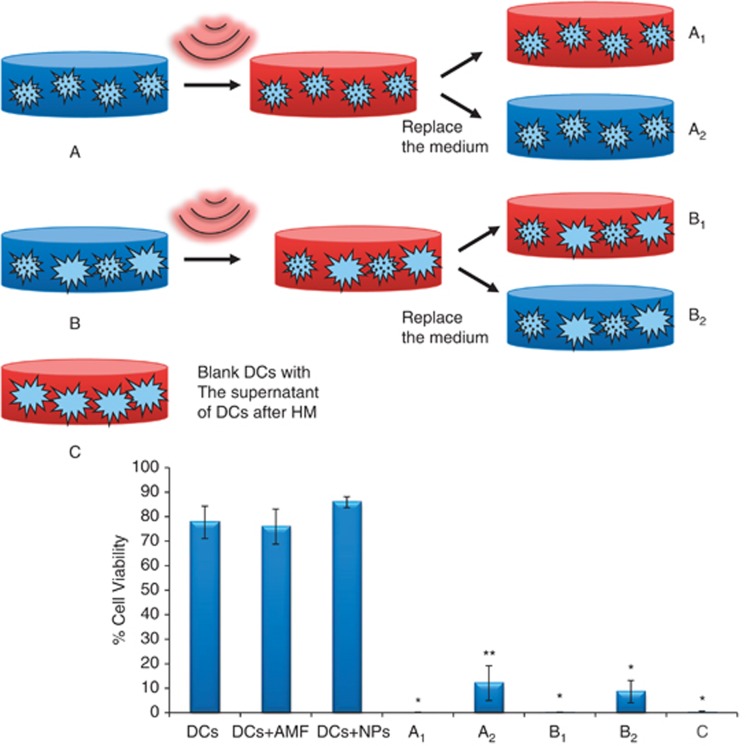
(Up) Scheme of the experiment proposed to test the toxicity of the supernatants of DCs loaded with NPs following AMF exposure. Experimental parameters for MH were as follows: 15 min, Hf=260 kHz and H=12.7 kA/m. The A and B samples were submitted to AMF and then divided into two halves. A_1_ and B_1_ were left intact, and for A_2_ and B_2_, the supernatant was replaced immediately after the AMF exposure with fresh medium. The C sample consisted of viable DCs that received the supernatants from the A and B samples and were incubated 30 min. (Down) Cell viability was measured by TB staining 15 min after AMF application. *N*=3. *Significant difference (*P*<0.0025) compared with the control sample (DCs). ** Significant difference (*P*<0.005) compared with the control sample

To test the potential influence of toxic agents in the supernatant on MNP-loaded DCs submitted to AMF culture, we performed the experiment depicted in Figure 3. This experiment used three types of samples (termed A, B and C). The first sample (A) corresponded with DCs incubated with 50 *μ*g Fe_3_O_4_/ml, which was divided into two parts following AMF exposure. One-half of the culture was left intact (A_1_), whereas the supernatant from the other half was immediately replaced by fresh culture medium following AMF exposure (A_2_). The second sample (B) consisted of a 1 : 1 mixture of DCs incubated with 50 *μ*g Fe_3_O_4_/ml and DCs incubated without MNPs, and the procedure was the same as that used for the first sample (B_1_ and B_2_). Owing to the previously mentioned intrinsic internalisation properties of the DCs, the samples contained ∼25% of MNP-loaded DCs and 75% of DCs without MNPs. The third sample (C) was formed using DCs that had never been in contact with either MNPs or AMF, and this sample received the supernatant that was removed from the A and B samples.

Following AMF exposure, the cell viability of all samples was analysed by trypan blue (TB) staining, and the results are shown in Figure 3. The results demonstrated that both the A_1_ and B_1_ samples experienced nearly 100% cell death, which confirmed the hypothesis that the DCs without MNPs died after the AMF exposure because they were in contact with MNP-loaded DCs. Following the addition of fresh medium, samples A2 and B2 demonstrated that a portion of the DCs remained alive, which is in agreement with our hypothesis concerning the toxicity of the supernatant. Furthermore, the reference DCs not placed in contact with MNPs and not submitted to AMF also died after receiving the supernatant removed from samples A and B.

## Conclusion

In conclusion, our results showed that only 50% of DCs in culture were able to internalise MNPs but that MNP-loaded DCs submitted to AMF released an as-yet-unidentified toxic agent(s) into the extracellular space that triggered the death of neighbouring cells. These results shed light on the mechanism responsible for cell death following MH treatment.

## Materials and Methods

For the study of internalisation by fluorescence-activated cell sorting (FACS) analysis, DCs cultures were setup as follows:^7, 12^ peripheral blood mononuclear cells were isolated from normal blood by density gradient (Ficoll Histopaque-1077, Sigma, Madrid, Spain). Cells were washed twice with PBS at 1200 r.p.m. and then centrifuged 10 min at 800 r.p.m. to avoid platelet contamination. Isolation of CD14+ cells was performed with magnetic beads (CD14 Microbeads, Miltenyi Biotec, Madrid, Spain) by positive immunoselection using the autoMACS Separator (Miltenyi). Positive CD14+ cells (10^6^ cells/ml) were cultured in RPMI 1640 (Sigma) with 10% FBS, 1% glutamine, 1% antibiotics and supplemented with interleukin (IL)-4 (25 ng/ml) and granulocyte-macrophage colony stimulating factor (25 ng/ml)(Bionova, Madrid, Spain), for 5 days at 37 °C. Every second day, medium was replaced by fresh medium containing the same concentration of ILs. The cells were collected on the fifth day, seeded into 24-well plates at a concentration of 10^6^ cells/ml and incubated overnight with different concentrations of fluorescent NPs (0, 10, 20, 50, 150, 200, 300 and 500 *μ*g/ml). The following day, the cells were washed three times by centrifugation with fresh medium and analysed using a FACSArea (Becton Dickinson and Company, Madrid, Spain). Data were analysed with FACSDiva software. The fluorescent NPs used in these experiments are commercially available from MicromodPartikeltechnologie GMBH, Rostock, Germany, Micromer-redF.

The MH experiments were performed in a 2 × 2 design, as described previously.^12^ In summary, this design consisted of two samples of DCs without MNPs and two samples of MNPs-loaded DCs. One of each group was submitted to AMF and the other one was leaved as controls to test the viability of the cell culture and the toxicity of the MNPs separately. The DCs were collected on the fifth day of culture and seeded into 12-well plates at a concentration of 10^6^ cells/ml; typically, 2 × 10^6^ cells were used for each sample. Following overnight incubation with MNPs at a concentration of 50 *μ*g Fe_3_O_4_/ml, the cells were washed as described above and resuspended in 500 *μ*l of fresh medium. AFM exposure (15 min, *f*=260 kHz and *H*=12.7 kA/m) was performed in a home-made device described elsewhere.^7, 12^ The sample designed to actually test the effect of AMF (i.e., the sample of MNPs-loaded DCs and submitted to AMF), was made in duplicate. One sample (A) consisted of 100% of DCs incubated with MNPs and the other one (B) was formed by mixing 50% of DCs incubated with MNPs and 50% reference DCs. Just immediately after the AFM exposure these samples were divided in two halves; A_1_ and B_1_ were leaved intact and the supernatant of A_2_ and B_2_ were replaced by fresh medium. Cell viability of these samples were measured 15 min following exposure by TB staining. The supernatant removed from A_2_ and B_2_ samples was placed on reference healthy viable non-magnetic-loaded DCs and incubated for 30 min. After that, cell viability was tested by TB.

Sample preparation for TEM and the acquisition images were performed as described elsewhere.^12^ Sample preparation for dual beam microscopy required the seed of 5 × 10^5^ cells per coverslip, followed by overnight incubation with MNPs. The samples were washed twice to remove excess MNPs and then fixed as previously described for TEM microscopy. The dehydration process was conducted using increasing concentrations of ethanol. Samples were then gold-coated and observed using a Nova 200TM NanoLab Dual Beam (FEI Company, Eindhoven, The Netherlands). The cells were cut into slices with a 10-nm thickness using the ion beam at a voltage of 30 kV and current of 10 pA and observed using the electron beam at a voltage of 5 kV and current of 0.4 nA.

## Abbreviations

AMF: alternating magnetic field
DCs: dendritic cells
FACS: fluorescence-activated cell sorting
IL-4: interleukin 4
MH: magnetic hyperthermia
MNPs: magnetic nanoparticles
NPs: nanoparticles
Rho: rhodamine
SEM: scanning electron microscopy
TB: trypan blue
TEM: transmission electron microscopy
